# What is the relationship between higher triglyceride and kidney function decline in adults?

**DOI:** 10.1080/0886022X.2021.1896549

**Published:** 2021-03-16

**Authors:** Zhifang Zhao, Lei Song, Jibin Yao

**Affiliations:** a Department of Medicine, Northwest Minzu University, Lanzhou, PR China; b Department of Surgical Oncology, Gansu Provincial Hospital, Lanzhou, P.R. China

Dear Editor,

We read with great interest the recently published study by Huang et al. [1] entitled “Elevated atherogenic index and higher triglyceride increase risk of kidney function decline: a 7-year cohort study in Chinese adults”.

Chronic kidney disease (CKD) is now recognized as a worldwide public health problem and the prevalence is high in the general adult population. A decline in the estimated glomerular filtration rate (eGFR) plays a role in the adverse outcomes and CKD development. The study enrolled 3712 Chinese participants, and 1.70% (*n* = 63) of them were developed major kidney function decline which was defined as a  ≥ 30% reduction in theeGFR from baseline after 7-year follow-up. The mean annual eGFR decline was 1.8 mL/min/1.73 m^2^. The article showed that a higher serum triglyceride level increased the risk of major kidney function decline in Chinese men with normal kidney function. The univariate and multivariable logistic regression models displayed that, TG was significantly associated with major kidney function decline in all participants (OR, 1.26, 95% CI, 1.10–1.43, *p* = 0.001). The association between higher TG and the prevalence of major kidney function decline was observed for Models 1 and 2 in Table 2 (OR, 1.28, 95% CI, 1.11–1.46, *p* < 0.001 and OR, 1.23, 95% CI, 1.06–1.43, *p* = 0.006, respectively). However, in unadjusted and adjustment 1 models, an elevated risk of major kidney function decline was observed with an increased TG (OR, 2.17, 95% CI, 1.17–4.04, *p* = 0.014 and OR, 2.26, 95% CI, 1.18–4.34, *p* = 0.014, respectively), while the fully adjusted model has no correlation in Table 3(OR, 1.74, 95% CI, 0.86–3.53, *p* = 0.123). I pay special attention to the results of this research because it caused me some confusion.

Firstly, an observational retrospective study conducted by Cao et al. [2] a total of 666 elderly from China with a baseline eGFR ≥ 60 mL/min/1.73 m^2^, and 6.01% (*n* = 40) developed low eGFR (<60 mL/min/1.73 m^2^) values, 11.11% (*n* = 74) showed reduced eGFR (>25%) at the end of 3-year follow-up. All renal outcomes were significantly worse in subjects with high TG (≥1.7 mmol/l) than at baseline. The logistic regression analyses showed that, TG > 1.7 mmol/L was associated with eGFR < 60 mL/min/1.73 m^2^ (OR, 2.44, 95% CI, 1.27–4.68, *p* = 0.007) in Model 1. Further adjustment for potential confounders factors, the association is slightly weakened in Model 2 and 3 (OR, 2.01, 95% CI, 1.00–4.04, *p* = 0.05 and OR, 1.71, 95% CI, 0.72–4.05, *p* = 0.221, respectively).

Secondly, Kang et al. [3] evaluated the relationship between the ratio of TG (mg/dl) to high-density lipoprotein cholesterol (HDL-C, mg/dl) and chronic kidney disease. 5,503 adult subjects from the Korean Ministry of Health and Welfare in 2005 were included. The overall prevalence of CKD which was defined as an eGFR below 60 mL/min/1.73 m^2^ was 9.0% (*n* = 495).The study cohort classified TG/HDL-C into 5 quintile groups (Q_1_ < 1.38, Q_2_1.38–2.01, Q_3_2.02–2.88, Q_4_2.89–4.49, Q_5_ ≥ 4.50), and showed that the OR for CKD in the Q_5_ (≥4.50) compared to the Q_1_ (< 1.38) was 3.20 (95% CI, 2.28–4.49, *p* = 0.001) by the logistic regression analysis. Even after adjustment for multiple covariates (Models 2 and 3), participants in the fifth quintile of TG/HDL-C were associated with increased risk of CKD (OR, 2.42, 95% CI, 1.63–3.60, *p* = 0.005 and OR, 2.15, 95% CI, 1.38–3.37, *p* = 0.036, respectively).

Finally, Rahman et al. [4] conducted a Chronic Renal Insufficiency Cohort (CRIC) study enrolled 3939 participants aged 21–74 years who were from seven clinical centers throughout the United States for 5-year follow-up. The results demonstrate that triglycerides were not independe- ntly predictive of progression of kidney disease (slope difference per 1 SD, 0.05, 95% CI, −0.06–0.15, *p* = 0.39). A recent two-sample mendelian randomization (MR) study reported that genetically higher triglyceride level had no association with eGFR < 60 mL/min/1.73 m^2^ (OR, 1.06, 95% CI, 0.97–1.62, *p* = 0.2) [5].

In conclusion, I think the inconsistence of study results may be caused by different ethnicities, different lifestyle, different genetic factors and different eGFR decline criterion as well as different study participants’ ages and exclusion criteria. The present study provides useful knowledge about the association between higher triglyceride and kidney function decline, but it does not give us sufficient data regarding TG level stratification. Further longitudinal, multicenter and well-conducted studies are needed to provide more evidence for designing treatment strategies for preventing the development and progression of CKD.

Zhifang Zhao and Lei Song *Department of Medicine, Northwest Minzu University, Lanzhou, PR China* Jibin Yao *Department of Surgical Oncology, Gansu Provincial Hospital, Lanzhou, P.R. China* 9binbin9@sina.com

## References

[1] Huang F, Wang L, Zhang Q, et al. Elevated atherogenic index and higher triglyceride increase risk of kidney function decline: a 7-year cohort study in Chinese adults. Ren Fail. 2021;43(1):32–39. 33307922 10.1080/0886022X.2020.1853569 PMC7745844

[2] Cao Y, Sun G, Liu R, et al. Plasma triglyceride levels and central obesity predict the development of kidney injury in Chinese community older adults. Renal Fail. 2019;41(1):946–953. 10.1080/0886022X.2019.1655451 PMC6807648 31599192

[3] Kang HT, Shim JY, Lee YJ, et al. Association between the ratio of triglycerides to high-density lipoprotein cholesterol and chronic kidney disease in Korean adults: the 2005 Korean National Health and Nutrition Examination Survey. Kidney Blood Press Res. 2011;34(3):173–179. 21502765 10.1159/000323895

[4] Rahman M, Yang W, Akkina S, et al. Relation of serum lipids and lipoproteins with progression of CKD: The CRIC study. CJASN. 2014;9(7):1190–1198. 24832097 10.2215/CJN.09320913 PMC4078958

[5] Lanktree MB, Thériault S, Walsh M, et al. HDL cholesterol, LDL cholesterol, and triglycerides as risk factors for CKD: a Mendelian randomization study. Am J Kid Dis. 2018;71(2):166–172. 28754456 10.1053/j.ajkd.2017.06.011

